# Clinical outcomes of a new diffractive trifocal intraocular lens with Enhanced Depth of Focus (EDOF)

**DOI:** 10.1186/s12886-016-0389-8

**Published:** 2016-11-29

**Authors:** Banu Torun Acar, Erkan Duman, Saban Simsek

**Affiliations:** 1 Haydarpasa Numune Education and Research Hospital, Ophthalmology Clinic, Kavakli sok. Menekse ap. No.22K.7 D.14 Caddebostan, Istanbul, Turkey; 2 Tuzla Government Hospital, Ophthalmology Clinic, Istanbul, Turkey

**Keywords:** Enhanced depth of focus, Intraocular lens, Trifocal

## Abstract

**Background:**

To evaluate the clinical outcomes after the implantation of a new trifocal diffractive intraocular lens (IOL) combined with Enhanced depth of focus (EDOF) technology.

**Methods:**

The study enrolled 80 eyes of 40 patients who underwent cataract surgery with bilateral implantation of a diffractive trifocal IOL (Reviol Tri-ED) designed with a combination of enhanced depth of focus. Mean age was 52.09 ± 11.32 years (range from 45 to 70 years). Uncorrected distance visual acuity (UDVA), corrected distance visual acuity (CDVA), uncorrected intermediate visual acuity (UIVA), corrected intermediate visual acuity (CIVA), uncorrected near visual acuity (UNVA), corrected near visual acuity (CNVA), keratometry (K), and manifest refraction spherical equivalent (MRSE) were evaluated pre- and postoperatively. The contrast sensitivity, defocus curves, and a questionnaire evaluating individual satisfaction were also estimated.

**Results:**

There was a significant improvement in UDVA, CDVA, UNVA, CNVA, CIVA postoperatively. The defocus curve confirmed good visual acuity also in the intermediate distance. The postoperative MRSE was ranged from −0.75 to 0.75 diopters. Contrast sensitivity also significantly improved postoperatively. The patient satisfaction was high.

**Conclusion:**

The new trifocal EDOF IOL provides visual improvement for far, intermediate, and near distances with a high level of visual quality and patient satisfaction.

## Background

Cataract, which is a treatable problem, is the leading cause of vision disorders and blindness all over the world [1]. Although aging is the primary cause of cataract, other factors associated with cataract formation include various diseases, trauma, medications and genetic predisposition. While the prevalence of visually significant cataract is about 2.5% at the age of 40–49 years, it increases with age and reaches to 68% before the age of 80 years [2]. The most frequent application in the surgical treatment of cataract is implantation of monofocal or multifocal intraocular lenses (IOLs) after removal of opacified lenses using phacoemulsification [2].

The use of IOLs aims at providing good and high-quality vision and reducing need for additional optical correction. Additionally, designs of monofocal IOLs allow either near or distance focus. In order to overcome this limitation, multifocal IOLs with refractive, diffractive, and the combination of both optical principles have been developed [3]. Multifocal IOLs can improve uncorrected near visual acuity (UNVA) and uncorrected distance visual acuity (UDVA). Nevertheless, different IOL models provide different levels of improvement for uncorrected intermediate visual acuity (UIVA). Various collateral effects such as halos, glare and loss of contrast sensitivity may be observed with the use of multifocal IOLs [4]. Many domestic and professional tasks including the use of computers require a good intermediate vision. While bifocal lenses cause difficulty in intermediate vision, trifocal lenses provide an increase in intermediate vision without compromising distance and near vision [3]. Trifocal IOL models have recently been introduced and clinical outcomes have been reported in the literature. Technical properties of a new lens (Acriva Reviol Tri-ED), particularly enhanced depth of focus property, seem encouraging; thus, we used it in a group of patients and wish the present the initial results, which may be followed by comparative studies in the future.

The present study aimed to evaluate visual acuities (distance, intermediate and near), refractive changes, contrast sensitivity, defocus curve, and postoperative satisfaction of a new diffractive trifocal IOL (Acriva Reviol Tri-ED).

## Subjects and methods

This prospective study comprised of bilateral cataract or presbyopia/pre-presbyopia suitable for refractive lens exchange patients who underwent routine phacoemulcification with diffractive trifocal IOL implantation between the periods of August 2014 and July 2015 at the Haydarpasa Numune Education and Research Hospital, Ophthalmology Clinic, Istanbul, Turkey. Informed consents of the patients were obtained. The study adhered to the tenets of the Declaration of Helsinki and was approved by the local ethics committee. Inclusion criteria were patients with bilateral cataract or presbyopia/pre-presbyopia suitable for refractive lens exchange and seeking for spectacle independence. Exclusion criteria were a history of glaucoma or retinal detachment, corneal disease, regular corneal astigmatism greater 0.75D, irregular corneal astigmatism, abnormal iris, macular degeneration or retinopathy, neurophthalmic disease, history of ocular inflammation and previous ocular surgery.

In the preoperative period, the following evaluations were performed: Distance (6 m, Early Treatment of Diabetic Retinopathy Study [ETDRS]), intermediate (80 cm) and near (40 cm) VA with and without correction, slit lamp examination, applanation tonometry, corneal topography (Sirius 3D, CSO, Italy), dilated fundus examination, optical biometry (IOLMaster version 4.3, Carl Zeiss Meditec AG, Germany) using SRK/T formula. The cataracts were graded using the LOCS III classification by the same examiner after slit-lamp examination [5].

All surgeries were performed by the same experienced surgeon (BTA) using a standard technique of sutureless microcoaxial 1.8–2.2 mm phacoemulsification. All incisions were made at the steep axis of the cornea. A 1.8 mm incision was made and 1.8 mm injector was used. In case of difficulty, the incision was extended up to 2.2 mm. After capsulorhexis creation and phacoemulsification, the IOLs were inserted into the capsular bag using the Acrijet Blue injector (VSY Biotechnology, Amsterdam, Netherlands) through the main incision. Postoperatively, all patients received the same treatment: a combination of an antibiotic and steroid agent.

The tests used in the preoperative period were also performed at the1^st^, 3rd and 6th months, except contrast sensitivity. Contrast sensitivity was evaluated preoperatively and at month 6, and measurement was performed under photopic (85 candelas [cd]/m2) conditions (CSV-1000, VectorVision, Ohio, USA). For the evaluation of the defocus curve, patients wore the correction providing the distance visual acuity in both eyes and the ETDRS charts were used at a distance of 4 m. Different levels of defocus were introduced in 0.5 D steps from +1.50 D to 4.00 D, and visual acuity values were recorded.

In order to evaluate patient satisfaction, the VFQ-14 (14-item Visual Function Questionnaire) was administered to the patients via e-mail at the postoperative 6th month. VFQ-14 questionnaire was sent via e-mail and patients were asked to respond within one week. Patients that did not respond until the specified deadline were called by phone and reminded about the questionnaire. Finally, all patients responded. To avoid patients being influenced, they answered the questions on their own. The questionnaire consists of 14 questions covering 14 aspects of visual function [6]. Each item was scored between 0 and 4 points, highest total point being 56. The degree of difficulty experienced while performing activities related to vision was assessed as no difficulty (4 points), a little difficulty (3 points), a moderate amount of difficulty (2 points), a great deal of difficulty (1 point), and unable to do the activity (0 point). The average of the points was calculated; higher points indicate a less difficulty in performing activities. In addition, all patients were questioned on spectacle need and photic phenomena during the 6th month visit.

**Fig. 1:**
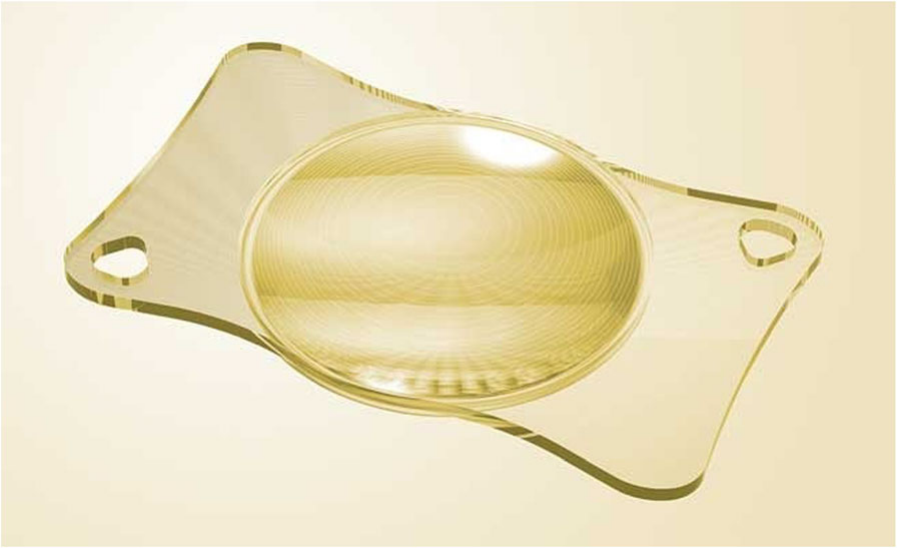
The new model of trifocal diffractive intraocular lens, Acriva Reviol Tri-ED

The Acriva Reviol Tri-ED is an IOL with a single piece diffractive trifocal EDOF design (Fig. 1). The characteristics of the IOL is presented on the Table 1.

**Table 1:** General IOL parameters [7]

Parameters	Reviol Tri-ED 611
Material	Hydrophobic surface, acrylic with 25% water content, blue filter
Optic size	6.00 mm
Optic design	Active-Diffractive Tri-ED
Haptic size	11.00 mm
Haptic Design	Plate Haptic
Haptic Angle	0°
Recommended Ac. A Constant	118.0
Recommended Op. A Constant	Srk-T: 118.3 – SRK-II: 118.5
Diopter Power Range	From 0.0 D to +32.00 D (0.50 D increments)
Refractive Index Dry	20 °C/35 °C 1.509/1.509 ± 0.002
Refractive Index Wet	20 °C/35 °C 1.462/1.462 ± 0.002
Lightdistribution (far%/intermediate%/near%)	44/28/28
Transmission Value (%)	89.1
Chromatic Aberration Control (Abbe number)	58
PCO prevention	360° sharp edge
Recommended Injector and Cartridge System	Acrijet

*IOL* intraocular lens, *D* diopter, *PCO* posterior capsule opacification

Product features are defined in manufacturer’s documents [7]. The EDOF feature of the lens provides a different advantage from the other available trifocal IOLs. Trifocal EDOF combination was created by changing height, width, interval and number of the diffractive rings. It’s a semi-apodized active diffractive trifocal structure is designed to reduce unwanted diffraction to increase optical quality with enhanced depth of focus vision. The entire optic dimeter covers 25 diffractive rings. This IOL has a trifocal anterior surface and provides an addition of 3.00 D for near and 1.50 D for intermediate at the IOL plane. Its design allocates 44% of light to distance, 28% to intermediate, and 28% to near for photopic and mesopic light condition; its overall efficiency of global light transmittance is 89.1%. The IOL is fully pupil diameters independent and provides adequate visual performance under all lighting conditions. It has a plate-haptic design with no haptic angulation with all enhanced 360-degree square edge to prevent posterior capsule opacification formation. It has spherical powers of 0.00 D to C32.00 D in 0.50 D increments and is implanted with a single-use injector through 2.2 mm incision.

### Statistical analysis

NCSS (Number Cruncher Statistical System) 2007&PASS (Power Analysis and Sample Size) 2008 Statistical Software (Utah, USA) programs were used for statistical analyses. In addition to the descriptive statistics (mean, standard deviation, median, frequency, ratio, minimum, maximum), quantitative data were evaluated also by Friedman Test for intragroup comparison and Wilcoxon signed-rank test for paired comparison of the parameters that were not distributed normally. Categorical variables were compared using chi-square test. The surgically induced astigmatism (SIA) was defined as the vector of change in corneal astigmatism between the preoperative and postoperative period. Corneal astigmatism data, obtained by corneal topography, were transformed into Cartesian (x and y) coordinates and surgically induced astigmatism (SIA) was calculated using a standard vector analysis. After the calculations were finished, the Cartesian coordinates were transformed back to the standard notation for astigmatism (cylinder and meridian). Statistical significance was evaluated at the levels of *p* < 0.01 and *p* < 0.05.

## Results

The present study included 40 patients who underwent bilateral trifocal IOL implantation. Patient characteristics are summarized in Table 2. The mean nuclear opalescence grade was 3.2 (range 1.2 to 4.1) for the 35 patients operated for cataract.

**Table 2:** Patient characteristics

Parameters	
Number of eyes	80
Right	40
Left	40
Gender, male/female	24/16
Age, years, mean ± SD, (range)	52.09 ± 11.32 (45–70)
Follow-up, months, mean ± SD	9.2 ± 2.1

*SD* standard deviation

### Visual acuity

**Fig. 2:**
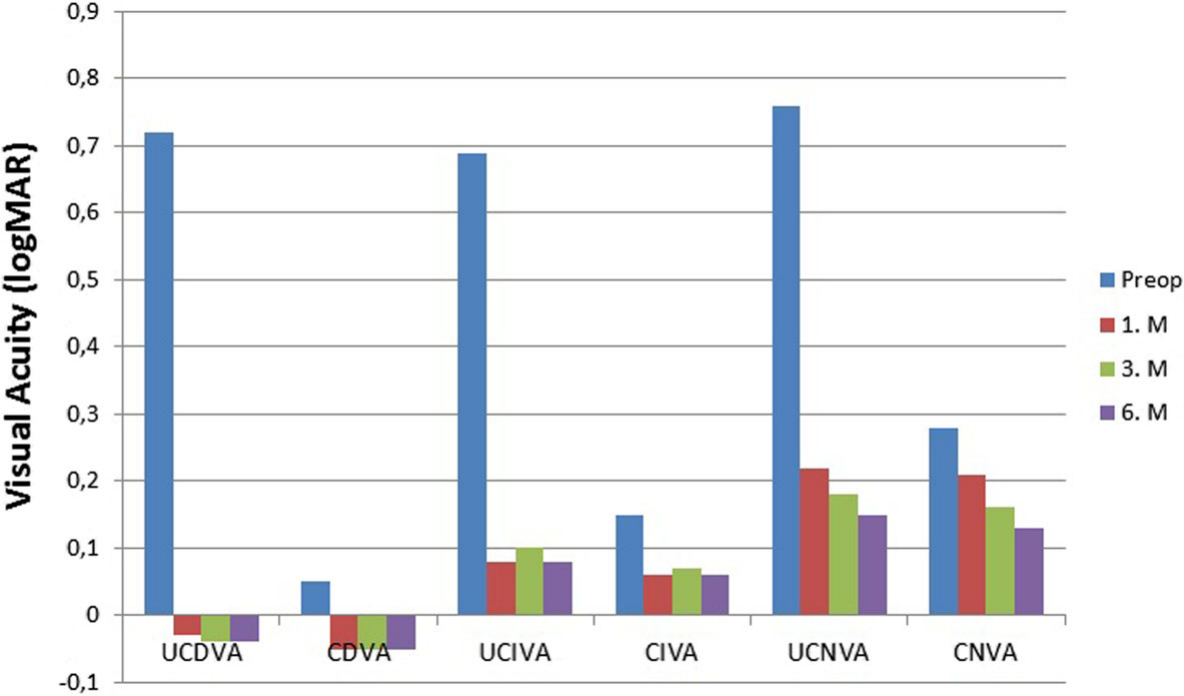
Visual acuity outcomes for distance (6 m), intermediate (80 cm), near (40 cm) distances during the whole period of follow up. (UDVA = uncorrected distance visual acuity; CDVA = corrected distance visual acuity; UIVA = uncorrected intermediate visual acuity; CIVA = corrected intermediate visual acuity; UNVA = uncorrected near visual acuity; CNVA = corrected near visual acuity)

Visual acuities over time are demonstrated in Table 3 and Fig. 2. Statistically significant difference was determined between preoperative values and postoperative 6th-month values of UDVA, UIVA and UNVA. Uncorrected variables showed significant improvement after surgery as compared to the preoperative values.

**Table 3:** LogMar visual acuity changes over time

Visual acuity	Preoperative	1 M	3 M	6 M	*P* value*
UDVA					
Mean ± SD	0,72 ± 0,20	−0,03 ± 0,08	−0,04 ± 0,08	−0,04 ± 0,08	0.001
Range	0,4–0,9	−0,2–0,2	−0,2–0,2	−0,2–0,2	
CDVA					
Mean ± SD	0,05 ± 0,18	−0,05 ± 0,05	−0,05 ± 0,05	−0,05 ± 0,08	0,018
Range	0,0–0,5	−0,2–0,2	−0,2–0,2	−0,2–0,2	
UNVA					
Mean ± SD	0,76 ± 0,16	0,22 ± 0,13	0,18 ± 0,13	0,15 ± 0,12	0,001
Range	0,4–1,4	−0,1–0,5	0,0–0,5	0,0–0,5	
CNVA					
Mean ± SD	0,28 ± 0,18	0,21 ± 0,12	0,16 ± 0,11	0,13 ± 0,04	0,340
Range	0,0–0,8	−0,1–0,5	0,0–0,3	0,0–0,4	
UIVA					
Mean ± SD	0,69 ± 0,18	0,08 ± 0,12	0,10 ± 0,10	0,08 ± 0,11	0,001
Range	0,1–1,2	−0,1–0,5	−0,1–0,4	−0,1–0,4	
CIVA					
Mean ± SD	0,15 ± 0,2	0,06 ± 0,07	0,07 ± 0,08	0,06 ± 0,10	0,120
Range	0,0–0,5	−0,1–0,4	−0,1–0,4	−0,1–0,4	

*UDVA* uncorrected distance visual acuity, *CDVA* corrected distance visual

*UNVA* uncorrected near visual acuity acuity, *CNVA* corrected near visual acuity

*UIVA* uncorrected intermediate visual acuity, *CIVA* corrected intermediate visual acuity, *SD* standard deviation, *M* month

*6 month vs. preoperative measurement

### Keratometry

The results of keratometry are demonstrated in Table 4. No significant difference was determined between preoperative values and postoperative 6th-month values in terms of the flattest meridian (Kf), steepest meridian (Ks) and keratometric astigmatism (Ks-Kf). According to vector analysis, the mean surgically induced astigmatism based on keratometry data was −0.23 ± 0.18 D (range, [−0.02]-[−1.00 D]).

**Table 4:** Refractive and keratometric changes over time

Parameters	Preoperative	1 M	3 M	6 M	*P* value*
Sphere, D					
Mean ± SD	0.40 ± 2.50	−0.10 ± 0.45	−0.00 ± 0.20	−0.02 ± 0.28	0.001
Range	−6.00, 3.50	−0.75, 0.75	−0.0, 0.50	−0.75, 0.50	
Cylinder, D					
Mean ± SD	−0.45 ± 0.36	0.35 ± 0.23	0.28 ± 0.13	0.28 ± 0.12	0.02
Range	−0.75, 0.00	−0.75, 0.0	0.50, 0.50	0.50, 0.00	
MRSE, D					
Mean ± SD	0.70 ± 2.28	0.24 ± 0.42	0.16 ± 0.35	0.12 ± 0.31	0.001
Range	−5.50, 3.25	−0.75, 0.75	−0.75, 0.50	−0.75, 0.75	
K2, D					
Mean ± SD	43.54 ± 1.52	43.46 ± 1.01	43.52 ± 1.12	43.48 ± 1.35	0.618
Range	40.43, 45.25	40.25, 45.48	40.21, 44.52	40.66, 45.51	
K1, D					
Mean ± SD	42.92 ± 1.28	42.86 ± 1.12	42.90 ± 1.34	42.88 ± 1.26	0,868
Range	39.75, 47.50	39.75, 45.00	39.75, 45.00	39.75, 45.00	
K2-K1, D					
Mean ± SD	0.62 ± 0.28	0.60 ± 0.25	0.62 ± 0.38	0.60 ± 0.38	0.612
Range	−0.18, 0.14	−0.15, 1.22	−0.09, 1.15	−0.05, 1.24	

*MRSE* manifest refraction spherical equivalent, *D* diopter, *K1* flat keratometry reading, *K2* steep keratometry reading, *M* month, *K2-K1* corneal cylinder, *SD* standard deviation

*6 month vs. preoperative measurement

### Refraction

Refractive changes of the patients over time are demonstrated in Table 4. Significant decrease was determined in the spherical and spherical equivalent values.

### Contrast sensitivity

**Fig. 3:**
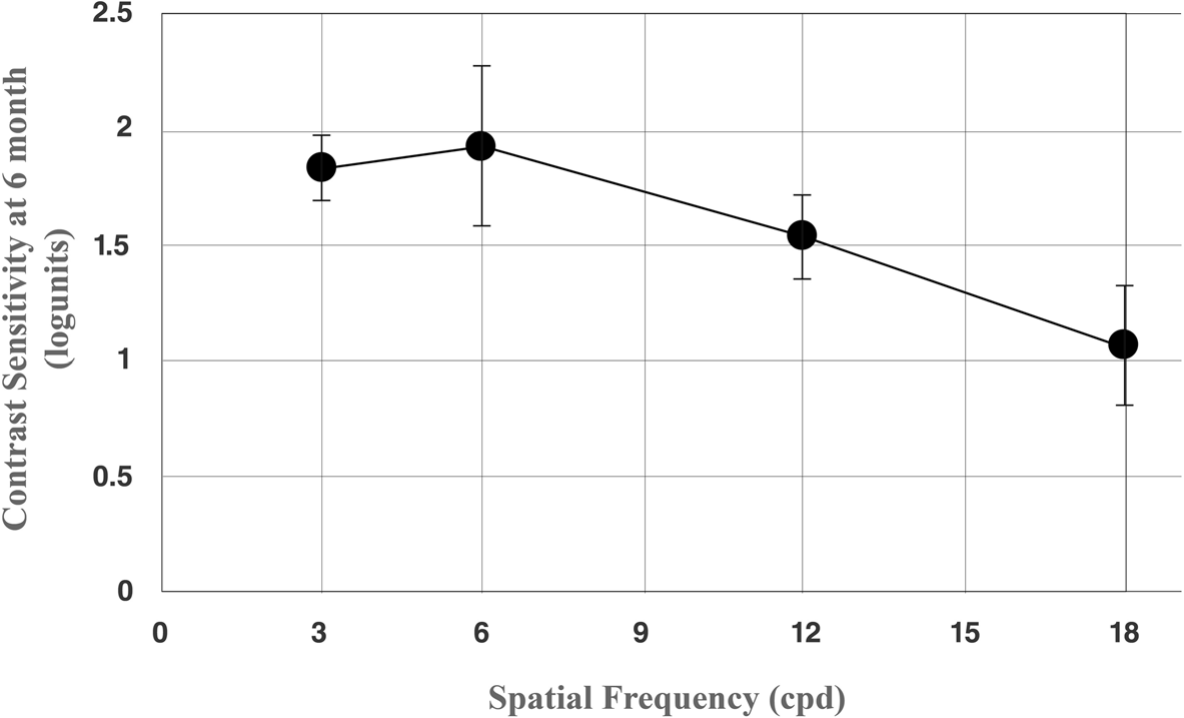
Mean contrast sensitivity outcomes under photopic conditions 6 months after the surgery with different spatial frequencies (cpd = cycles per degree)

The results of contrast sensitivity over time at 4 different spatial frequencies are depicted in Fig. 3. From 1 month to 6 months postoperatively, there was a slight but significant improvement in contrast sensitivity at 3 cpd (spatial frequency). The mean contrast sensitivity changed from 1.78 ± 0.20 log units to 1.83 ± 0.11 log units (*p* = 0.023). There was no significant improvement in contrast sensitivity at 6 cpd, 12 cpd, and 18 cpd between 1 month and 6 months postoperatively.

### Defocus curve

**Fig. 4:**
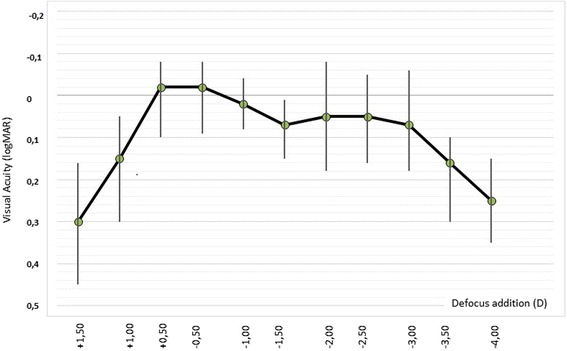
Defocus curve of Acriva Reviol Tri-ED intraocular lens

The mean visual acuities and their standard deviations for different defocus values are demonstrated in Fig. 4. In the present study, defocus curve obtained by trifocal IOL showed a tendency of flattening different from the typical M-shape observed by bifocal IOL. VA was preserved particularly in intermediate distances.

### Patient satisfaction

The results of the questionnaire applied at the postoperative 6th month are summarized in Table 5. More than 90% of the patients reported no difficulty in performing the daily activities related to vision. The average total score (sum of all questions) was 54.6, 55.1, and 51.0 over maximum score of 56 for the whole study group (*n* = 40), patients operated for cataract (*n* = 35), and patients operated for presbyopia/pre-presbyopia (*n* = 5), respectively. When item-based responses are considered, 95% (464/490) of total questions were scored 4 (no difficulty) in the cataract group, whereas the corresponding figure was 74% (52/70) in the presbyopia/pre-presbyopia group (*p* < 0.001), indicating higher satisfaction in cataract patients. In addition, none of the patients complained of photic phenomena and were all spectacle-free at the 6th month visit.

**Table 5:** The results of VFQ-14 Questionnaire performed to assess the patient satisfaction regarding the activities related to vision at the postoperative 6th month

Questions	Score	No difficulty	A little of difficulty	Moderate amount of difficulty	A great deal of difficulty	Unable to do
	Mean ± SD	*n* (%)	*n* (%)	*n* (%)	*n* (%)	*n* (%)
Reading small print	3.85 ± 042	35 (87.5)	4 (10.0)	1 (2.5)	0 (0.0)	0 (0.0)
Reading normal newsprint	3.95 ± 0.22	38 (95.0)	2 (5.0)	0 (0.0)	0 (0.0)	0 (0.0)
Reading large newsprint	3.97 ± 0.15	39 (97.5)	1 (2.5)	0 (0.0)	0 (0.0)	0 (0.0)
Recognizing faces at a distance	3.85 ± 0.48	36 (90.0)	2 (5.0)	2 (5.0)	0 (0.0)	0 (0.0)
Going downstairs	3.97 ± 0.15	39 (97.5)	1 (2.5)	0 (0.0)	0 (0.0)	0 (0.0)
Reading street signs	3.95 ± 0.22	38 (95.0)	2 (5.0)	0 (0.0)	0 (0.0)	0 (0.0)
Sewing, doing delicate manual work	3.80 ± 0.51	34 (85.0)	4 (10.0)	2 (5.0)	0 (0.0)	0 (0.0)
Reading mail, bills accurately	3.90 ± 0.37	37 (92.5)	2 (5.0)	1 (2.5)	0 (0.0)	0 (0.0)
Playing cards	3.95 ± 0.22	38 (95.0)	2 (5.0)	0 (0.0)	0 (0.0)	0 (0.0)
Going out to movies, plays, sporting events	3.95 ± 0.22	38 (95.0)	2 (5.0)	0 (0.0)	0 (0.0)	0 (0.0)
Cooking	3.90 ± 0.30	36 (90.0)	4 (10.0)	0 (0.0)	0 (0.0)	0 (0.0)
Watching television	3.95 ± 0.22	38 (95.0)	2 (5.0)	0 (0.0)	0 (0.0)	0 (0.0)
Driving on day	3.87 ± 0.40	36 (90.0)	3 (7.5)	1 (2.5)	0 (0.0)	0 (0.0)
Driving at night	3.75 ± 0.66	34 (85.0)	3 (7.5)	2 (5.0)	1 (2.5)	0 (0.0)

*SD* standard deviation

(no difficulty - 4 points; a little of difficulty - 3 points; moderate amount of difficulty – 2 points; a great deal of difficulty - 1 point; unable to do - 0)

### Complications

No serious complication (iridodialysis, posterior capsule perforation, etc.) occurred during the surgery; refractive surprise, postoperative macular edema and posterior capsule opacity (PCO) were encountered during follow-up period.

## Discussion

Recently, it is possible to obtain more successful outcomes in the treatment of patients with cataract with the developments in phacoemulsification techniques and IOL technology. Providing high-quality VA and best levels of spectacle independence for near, intermediate and distance vision has been the primary aim after multifocal IOL implantation [8, 9]. With the use of bifocal IOL implantation, successful outcomes have been obtained in distance and near vision along with increased quality of life and patient satisfaction; however, intended level of improvement could not be achieved in intermediate VA or different outcomes have been obtained depending on characteristics of lens or on patient selection [10–19]. In addition, some of the patients having bifocal IOL implantation complain about certain functional disorders such as haloes or rings around lights, glare and photopsia [20, 21].

Trifocal IOLs, which have three focal spots, have been introduced into use to overcome the problems experienced with bifocal lenses. It has been reported that the use of trifocal IOLs significantly improves intermediate VA without impairing near and distance vision [22, 23]. This also enhances patient satisfaction with its favorable effects on quality of life [22, 23].

Studies conducted on different commercial models of trifocal IOLs have reported good distance, intermediate and near VAs. In their study, Carballo-Alvarez et al. [24] performed FineVision trifocal IOL implantation in 44 eyes of 22 patients with cataract and reported that a full range of adequate vision was achieved, contrast sensitivity was satisfactory, and there were no significant adverse photic phenomena after implantation. Sheppard et al. [25] obtained good distance VA and near and intermediate visual function with the use of FineVision trifocal IOL. In the patients (54 eyes of 27 patients) who underwent AT LISA trifocal IOL implantation following phacoemulsification, Kohnen et al. [26] reported good distance, intermediate and near VAs (0.10 logMAR or better), a high patient satisfaction, and a high spectacle independence at the postoperative 3rd month. Kretz et al. [27] reported significant improvements in UDVA, UIVA, UNVA, and CDVA and better binocular outcomes as compared with monocular outcomes in 100 eyes of 50 patients who underwent AT LISA IOL implantation following cataract surgery.

In the present study, Acriva Reviol Tri-ED IOL was implanted in 80 eyes of 40 patients. The UDVA, UIVA and UNVA logMAR values were determined as 0.72 ± 0.20, 0.69 ± 0.18 and 0.76 ± 0.16, respectively, in the preoperative period and as −0.04 ± 0.08, 0.08 ± 0.11 and 0.15 ± 0.12, respectively, at the postoperative 6th month. These improvements in distance, intermediate and near VAs were found to be significant (*p* = 0.001 for each). The preoperative spherical equivalent refraction was 0.70 ± 2.28 D and a significant decrease to a value of 0.12 ± 0.31 D occurred at the postoperative 6th month. These results suggested that the trifocal IOL used in the present study was very effective. In their study, Vryghem and Heireman [28] implanted FineVision trifocal IOL in 50 eyes of 25 patients and reported binocular UDVA, UIVA and UNVA to be −0.04 ± 0.09, −010 ± 0.15 and 0.02 ± 0.06 logMAR, respectively, at the postoperative 6th month. In their study performed on 94 eyes of 47 patients, Cochener et al. [29] reported binocular UDVA, UIVA and UNVA as 0.02 ± 0.09, 005 ± 0.08 and 0.00 ± 0.04 logMAR, respectively at the 6th month following FineVision trifocal IOL implantation. Jonker et al. [30] compared the Finevision Micro F trifocal IOL with the Acrysof Restor IQ C3.0 D bifocal IOL in their randomized prospective study and indicated that better defocus curve was obtained by trifocal IOL in the intermediate distance. They also reported that the mean binocular UDVA, UIVA, and UNVA were 0.01 ± 0.11 logMAR, 0.32 ± 0.15 logMAR, and 0.15 ± 0.13 logMAR, respectively, in the patients implanted with trifocal IOL (30 eyes of 15 patients) at the postoperative 6th month. Kretz et al. [31] reported a binocular UDVA of 0.00 logMAR or better and a binocular UIVA of 0.10 logMAR or better in all patients undergoing AT LISA trifocal IOL implantation (76 eyes of 38 patients), and a binocular UNVA of 0.10 logMAR or better in 85% of the patients at the postoperative 3 months. Mojzis et al. [32] conducted a study in the patients (120 eyes of 60 patients) who underwent cataract surgery with trifocal AT LISA IOL implantation; and followed the patients for a postoperative period of 12- month. They reported that a complete and stable visual restoration and good levels of visual quality were achieved with the use of trifocal IOL during the follow-up period.

In the present study, acceptable changes were observed postoperatively in the keratometric parameters as compared with the preoperative period. This finding was consistent with the results of other studies performed with trifocal IOLs [22, 27]. It seems that steep meridian incision may not have clinically relevant flattening effects and small incision size may account for this outcome.

In the present study, the best levels of contrast sensitivity were achieved at lower (3 cpd) spatial frequencies. Likewise, Vryghem and Heireman [28] and Kretz et al. [31] also achieved the highest level of contrast sensitivity at 3 cpd. In the studies conducted by Mojzis et al. [22] and Sheppard et al. [25], the contrast sensitivity curve revealed that the patients had high sensitivity to medium (6 cpd) spatial frequencies. However, in this study, no change was observed at other spatial frequencies. Probably absence of a negative change at 6, 12 and 18 cpd may also be interpreted as clinically relevant, possibly indicating the absence of posterior capsular opacification.

In the present study, evaluation of the defocus curve obtained at the postoperative 6th month revealed that intermediate VA was also effectively improved in addition to near and distance VAs. According to the results of the questionnaire performed during the follow-up period, most of the patients had no difficulty in performing many activities related to vision and thus were satisfied with the results.

We believe that the outcomes we achieved support the idea that EDOF elements can be valuable option to be used as IOLs to restore the imaging ability of the pseudophakic eyes. Using this new design concept of IOLs promises an expanded depth of field without the drawbacks associated with a multifocal visual system. All our patients achieved spectacle independence without an incidence of photic phenomena, such as halos and glare at 6 month-follow up.

Several EDOF-related technical features of Reviol Tri-ED seem to provide some potential advantages: i) real trifocal structure at both center and periphery in contrast to other available trifocal IOLs (e.g. Finevision is a combination of two bifocal patterns and ATLisa has trifocal structure at the center but bifocal at the outer zone); ii) high modular transfer function (MTF) values for transitions, aiding seamless continuous vision; iii) the amount of light reaching retina is high and the ratios of light distributed to far, near and intermediate sights are similar resulting in better light utilization (other IOLs send more light for far vision); iv) intermediate and near sight additions are different thus potentially providing better intermediate sight (80 cm); v) pupil-independent due to semi-apodization feature; vi) higher Abbe value than other IOLs thus providing better chromatic aberration control to prevent halo glare [33], vii) diffractive surface transitions zones are smooth, possibly preventing halo-glare and low contrast sensitivity. However, although these features and findings of this study are encouraging, further comparative studies with other trifocal IOLs are warranted to examine whether these characteristics translate into better clinical outcomes.

The limitations of our study are small number of eyes included, also a reading speed is an important indicator of near visual performance which was not estimated in the current study. This might be also a limitation in terms of assessing the functional vision.

## Conclusions

Acriva Reviol Tri-ED used in the present study, a novel trifocal IOL, appears to be a new option in overcoming the problems experienced with bifocal lenses owing to its maximum light energy transmission, and tolerability. It is able to provide an effective distance, intermediate, and near visual acuities after cataract surgery, with high level of visual improvement, and patient satisfaction.
